# Negative impact of corticosteroid use on outcome in patients with advanced BTCs treated with cisplatin, gemcitabine, and durvalumab: A large real‐life worldwide population

**DOI:** 10.1002/ijc.70009

**Published:** 2025-07-11

**Authors:** Federica Lo Prinzi, Francesca Salani, Silvia Camera, Mario Domenico Rizzato, Anna Saborowski, Lorenzo Antonuzzo, Federico Rossari, Tomoyuki Satake, Frederik Peeters, Tiziana Pressiani, Jessica Lucchetti, Jin Won Kim, Oluseyi Abidoye, Ilario Giovanni Rapposelli, Chiara Gallio, Stefano Tamberi, Fabian Finkelmeier, Guido Giordano, Pircher Chiara, Hong Jae Chon, Chiara Braconi, Aitzaz Qaisar, Alessandro Pastorino, Florian Castet, Emiliano Tamburini, Changhoon Yoo, Alessandro Parisi, Anna Diana, Mario Scartozzi, Gerald W. Prager, Antonio Avallone, Marta Schirripa, Il Hwan Kim, Lukas Perkhofer, Ester Oneda, Monica Verrico, Nuno Couto, Jorge Adeva, Stephen L. Chan, Gian Paolo Spinelli, Nicola Personeni, Ingrid Garajova, Maria Grazia Rodriquenz, Silvana Leo, Cecilia Melo Alvim, Ricardo Roque, Mariam Grazia Polito, Emanuela Di Giacomo, Giovanni Farinea, Linda Bartalini, Giada Grelli, Antonio De Rosa, Daniele Lavacchi, Masafumi Ikeda, Jeroen Dekervel, Monica Niger, Rita Balsano, Giuseppe Tonini, Minsu Kang, Giulia Tesini, Alessandra Boccaccino, Vera Himmelsbach, Matteo Landriscina, Selma Ahcene Djaballah, Tanios Bekaii‐Saab, Lorenzo Fornaro, Gianluca Masi, Arndt Vogel, Sara Lonardi, Margherita Rimini, Lorenza Rimassa, Andrea Casadei‐Gardini

**Affiliations:** ^1^ Operative Research Unit of Medical Oncology Fondazione Policlinico Universitario Campus Bio‐Medico Rome Italy; ^2^ Unit of Medical Oncology 2 Azienda Ospedaliero‐Universitaria Pisana Pisa Italy; ^3^ Department of Oncology Vita‐Salute San Raffaele University, IRCCS San Raffaele Scientific Institute Hospital Milan Italy; ^4^ Department of Oncology Veneto Institute of Oncology IOV – IRCCS Padua Italy; ^5^ Hannover Medical School Hannover Germany; ^6^ Clinical Oncology Unit Careggi University Hospital Florence Italy; ^7^ Department of Experimental and Clinical Medicine University of Florence Florence Italy; ^8^ Department of Hepatobiliary and Pancreatic Oncology National Cancer Center Hospital East Kashiwa Japan; ^9^ Digestive Oncology University Hospitals Leuven Leuven Belgium; ^10^ Department of Translational Research and New Technologies in Medicine and Surgery University of Pisa Pisa Italy; ^11^ Medical Oncology and Hematology Unit Humanitas Cancer Center, IRCCS Humanitas Research Hospital Milan Italy; ^12^ Division of Hematology/Medical Oncology, Department of Internal Medicine Seoul National University Bundang Hospital, Seoul National University College of Medicine Seongnam‐si Republic of Korea; ^13^ Department of Internal Medicine Mayo Clinic Phoenix Arizona USA; ^14^ Department of Medical Oncology IRCCS Istituto Romagnolo per lo Studio dei Tumori (IRST) “Dino Amadori” Meldola Italy; ^15^ Medical Oncology Santa Maria delle Croci Hospital, Ravenna AUSL Romagna Italy; ^16^ Medical Clinic 1, Department of Gastroenterology University Hospital Frankfurt Frankfurt am Main Germany; ^17^ Unit of Medical Oncology and Biomolecular Therapy Policlinico Riuniti Foggia Italy; ^18^ Department of Medical and Surgical Sciences University of Foggia Foggia Italy; ^19^ Department of Medical Oncology Fondazione IRCCS Istituto Nazionale dei Tumori Milan Italy; ^20^ Division of Medical Oncology, Department of Internal Medicine CHA Bundang Medical Center, CHA University School of Medicine Seongnam South Korea; ^21^ Beatson West of Scotland Cancer Centre, CRUK University of Glasgow (School of Cancer Sciences) Glasgow Scotland, UK; ^22^ Medical Oncology Unit 1 IRCCS Ospedale Policlinico San Martino Genoa Italy; ^23^ Gastrointestinal and Endocrine Tumor Unit, Vall d'Hebron Institute of Oncology (VHIO) Hospital Universitari Vall d'Hebron, Vall d'Hebron Barcelona Hospital Campus Barcelona Spain; ^24^ Oncology Department and Palliative Care Cardinale Panico Tricase City Hospital Tricase Italy; ^25^ ASAN Medical Center University of Ulsan College of Medicine Seoul South Korea; ^26^ Clinica Oncologica e Centro Regionale di Genetica Oncologica Università Politecnica delle Marche, Azienda Ospedaliero‐Universitaria delle Marche Ancona Italy; ^27^ Oncology Unit Ospedale del Mare Naples Italy; ^28^ Medical Oncology University and University Hospital Cagliari Italy; ^29^ Department of Medicine I, Clinical Division of Oncology Medical University Vienna Austria; ^30^ Clinical Experimental Abdominal Oncology Unit Istituto Nazionale Tumori‐IRCCS Fondazione G. Pascale Naples Italy; ^31^ Medical Oncology Unit, Department of Oncology and Hematology Belcolle Hospital Viterbo Italy; ^32^ Division of Oncology, Department of Internal Medicine Haeundae Paik Hospital, Inje University College of Medicine Busan Republic of Korea; ^33^ Internal Medicine 1 University Hospital Ulm Ulm Germany; ^34^ Institute of Molecular Oncology and Stem Cell Biology Ulm University Hospital Ulm Germany; ^35^ Dipartimento di Oncologia Medica Fondazione Poliambulanza Brescia Italy; ^36^ UOC Oncologia A, Department of Hematology, Oncology and Dermatology Policlinico Umberto I University Hospital, Sapienza University of Rome Rome Italy; ^37^ Digestive Unit, Champalimaud Clinical Centre Champalimaud Research Centre Lisbon Portugal; ^38^ Department of Medical Oncology Hospital Universitario 12 de octubre Madrid Spain; ^39^ State Key Laboratory of Translational Oncology, Department of Clinical Oncology Sir YK Pao Centre for Cancer, Hong Kong Cancer Institute, Prince of Wales Hospital, The Chinese University of Hong Kong Hong Kong China; ^40^ UOC Oncologia Territoriale Polo Pontino, La Sapienza Università Di Roma Latina Italy; ^41^ Medical Oncology Unit P.O. Manerbio – ASST Garda Brescia Italy; ^42^ Medical Oncology Unit University Hospital of Parma Parma Italy; ^43^ Oncology Unit Fondazione IRCCS “Casa Sollievo della Sofferenza” San Giovanni Rotondo Italy; ^44^ Division of Oncology Vito Fazzi Hospital Lecce Italy; ^45^ Medical Oncology Department Hospital de Santa Maria, Centro Hospitalar Universitário Lisboa Norte Lisbon Portugal; ^46^ Portuguese Institute of Oncology of Coimbra Coimbra Portugal; ^47^ Department of Oncology University of Turin, San Luigi Hospital Turin Italy; ^48^ Department of Surgery, Oncology and Gastroenterology University of Padua Padua Italy; ^49^ Department of Biomedical Sciences Humanitas University Milan Italy; ^50^ Department of Medicine and Surgery Università Campus Bio‐Medico di Roma Rome Italy

**Keywords:** biliary tract cancers, cisplatin, gemcitabine, and durvalumab, concomitant medications

## Abstract

In recent years, there has been increasing interest in the possible prognostic impact of concomitant medications in patients with cancer treated with immunotherapy combinations. This real‐world analysis aims to evaluate the impact of concomitant medications on survival outcomes in patients with advanced biliary tract cancer (BTCs) treated with cisplatin, gemcitabine and durvalumab (CGD) therapy. The study cohort included patients with a diagnosis of advanced BTCs who were taking concomitant medications for their comorbidities before the start of CGD. The primary objectives were overall survival (OS) and progression‐free survival (PFS). The initial population consisted of 666 patients, who were retrospectively collected from 41 sites in 12 countries. Data on concomitant medications were available for 493 patients. After a median follow‐up of 8.8 months (95% CI: 7.8–9.8), patients who did not take steroids (prednisone >10 mg/day or equivalent) or nonsteroidal anti‐inflammatory drugs (NSAIDs) and opioids, before the start of CGD, had longer OS and PFS in univariate analysis. The multivariate analysis confirmed longer OS for patients who did not take steroids. Patients who did not take steroids had an OS of 14.8 months (95% CI: 13.1–29.1) versus 5.0 months (95% CI: 2.14–11.32) of patients who took prednisone >10 mg/day or equivalent. No differences were reported in terms of overall response rate (ORR), disease control rate (DCR) (*p* = 1.0 and *p* = .16, respectively), and safety profile between the two groups. Our analysis suggests that patients who did not receive steroids before the start of GCD had longer survival and highlighted the relevance of balancing concomitant medications and chemoimmunotherapy.

## What's new?

In recent years, the possible prognostic impact of concomitant medications in patients with cancer treated with immunotherapy combinations has generated increasing interest. This real‐world analysis evaluated the impact of concomitant medications on survival outcomes in patients with advanced biliary tract cancer treated with cisplatin, gemcitabine and durvalumab therapy. The findings indicate that patients who did not receive steroids (prednisone at a dose > 10 mg/day or its equivalent) before starting chemoimmunotherapy experienced longer survival than those who did receive steroids. The study highlights the relevance of balancing concomitant medications and chemoimmunotherapy in patients with cancer.

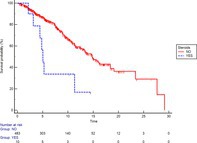

**Abbreviations**
AEs: adverse events
BTCs: biliary tract cancers
CGD: cisplatin, gemcitabine and durvalumab
CR: complete response
CI: confident interval
DCR: disease control rate
DFS: disease free survival
eCCA: extrahepatic cholangiocarcinoma
GBC: gallbladder cancer
HR: hazard ratio
iCCa: intrahepatic cholangiocarcinoma
NLR: neutrophil to lymphocyte ratio
NSAIDs: nonsteroidal anti‐inflammatory drugs
ORR: objective response rate
OS: overall survival
PD: progression disease
PFS: progression‐free survival
PR: partial response
PS: performance status
SD: stable disease

## 1 INTRODUCTION

Biliary tract cancers (BTCs) are a heterogeneous group of tumors of the biliary tree, including gallbladder cancer (GBC), intrahepatic (iCCA), and extrahepatic (distal, peri‐hilar), (eCCA) cholangiocarcinoma.^1^, ^2^, ^3^ Despite BTCs being considered rare tumors, their incidence has increased worldwide in the last decade.^4^ BTCs have a poor prognosis, with an estimated 5‐year overall survival (OS) rate of <20% when considering all stages.^4^, ^5^ New strategies have emerged for patients with advanced BTCs, like immunotherapy and molecularly targeted therapy in recent years.^6^, ^7^ Two first‐line standards of care are available thanks to the survival benefit of the combination of durvalumab or pembrolizumab with cisplatin/gemcitabine over chemotherapy alone shown in the TOPAZ‐1^8^, ^9^, ^10^ and KEYNOTE‐966^11^, ^12^ phase 3 studies. In the second and following lines, in addition to chemotherapy,^13^ targeted agents have shown their efficacy in phase 2 and 3 trials, providing new treatment options for patients with BTCs.^14^, ^15^, ^16^

Patients with cancer often receive several concomitant medications for their comorbidities, for adverse events caused by systemic therapy, or for symptoms related to oncological disease. The most common concomitant medications for previous comorbidities include antihypertensives, statins, and anticoagulants for cardiovascular disorders.^17^ Steroids and antiemetics are frequently prescribed to manage adverse events, while analgesics (NSAIDs and/or opioids) are used to relieve pain associated with cancer.

One of the most studied non‐oncologic drugs in gastrointestinal cancers is acetylsalicylic acid, recognized for its protective role against gastrointestinal cancers, especially colorectal cancer.^18^ Recently, it has also been observed to reduce the risk of hepatobiliary cancers.^19^, ^20^ Casadei Gardini et al. suggested that vitamin D intake may enhance disease‐free survival (DFS) in patients with BTCs after surgery and that starting metformin after chemotherapy (without immunotherapy) may improve outcomes in advanced disease stages.^21^ No data on other concomitant medications are available in the literature.

Given the limited available data on the use of concomitant medications in patients with advanced BTCs treated with chemotherapy, and particularly in combination with immunotherapy, the aim of this study is to evaluate the impact of basal concomitant medications on clinical outcomes in patients with advanced BTCs treated with cisplatin, gemcitabine, and durvalumab in a large real‐life worldwide population.

## 2 MATERIALS AND METHODS

### 2.1 Study population

The study population included patients with unresectable, locally advanced, or metastatic BTCs, including iCCA, eCCA, and GBC who were taking concomitant medications for their comorbidities before the start of cisplatin, gemcitabine and durvalumab (CGD). Data were retrospectively collected from 41 sites in 12 countries (Italy, Germany, Austria, Spain, Belgium, Portugal, United Kingdom, United States of America, Republic of Korea, China, Hong Kong Special Administrative Region of China, and Japan).

Patients were treated with CGD administered intravenously on a 21‐day cycle for up to eight cycles. Durvalumab (1500 mg) was administered on day 1 of each cycle, in combination with gemcitabine (1000 mg/m^2^) and cisplatin (25 mg/m^2^), which were administered on days 1 and 8 of each cycle. After completion of gemcitabine and cisplatin, durvalumab monotherapy (1500 mg) was administered every 4 weeks until clinical or imaging disease progression or unacceptable toxicity was reached. The concomitant medications were categorized as follows: beta‐blockers, ACE inhibitors, antihypertensives, metformin, insulin, pancrelipase, anticoagulants/antiplatelets, anxiolytics/antidepressants, proton pump inhibitors, levothyroxine, diuretics, statins, acetylsalicylic acid, vitamin D, ursodeoxycholic acid, steroids (prednisone >10 mg/day or equivalent), and analgesics (NSAIDs and/or opioids).

### 2.2 Statistical analysis

This analysis aims to assess the impact of concomitant medications on survival outcomes (OS and progression‐free survival [PFS]) in patients treated with cisplatin, gemcitabine, and durvalumab. OS was defined as the time from the beginning of first‐line therapy to death from any cause. PFS was defined as the time from the beginning of the first line of therapy to disease progression or death. OS was estimated by the Kaplan–Meier method, and curves were compared by the log‐rank test. Unadjusted and adjusted hazard ratios (HRs) by baseline characteristics were calculated using the Cox proportional hazards model. A *p*‐value <.05 was considered statistically significant.

Treatment response was evaluated by computed tomography and categorized as complete response (CR), partial response (PR), stable disease (SD), or progressive disease (PD) by local review according to Response Evaluation Criteria in Solid Tumors (RECIST) 1.1. Overall response rate (ORR) was defined as the proportion of patients who achieved CR or PR. Disease control rate (DCR) was defined as the proportion of patients who achieved CR, PR, or SD.

Adverse events (AEs) were graded according to the National Cancer Institute Common Terminology Criteria for Adverse Events, version 5.0.

MedCalc package (MedCalc version 16.8.4) was used for statistical analysis.

## 3 RESULTS

### 3.1 Patients

The initial population consisted of 666 patients with locally advanced unresectable or metastatic BTCs. Data on concomitant medications were available for 493 patients (74%), and all of them received concomitant medications. Data on concomitant medications for the remaining 173 patients (26%) are not available. Ten patients received steroids at the dose of prednisone >10 mg/day or equivalent. Patient characteristics and type of concomitant medications are summarized in Table 1. Exactly 261 patients (52.9%) were male with a median age of 70 years (range 31–91) and an ECOG performance status (PS) of 0–1. The majority of patients (78.4%) had metastatic disease. Most patients (357 patients; 72.4%) had no drainage or biliary stent, and 281 patients (56.9%) had normal weight. Median baseline CA 19‐9 levels were 111 IU/mL (range 0.60–400,000). A total of 319 patients (64.7%) had elevated CA 19‐9, and 244 of patients (49.3%) had NLR ≥3.

**TABLE 1:** Patient characteristics.

Characteristic	Patients; *N* (%); *N* = 493
Gender	
Male	261 (52.9)
Female	232 (47.0)
Age	70 (range 31–91)
>70	234 (47.4)
≤70	259 (52.5)
Primary tumor site	
Intrahepatic	273 (55.3)
Extrahepatic	121 (24.5)
Gallbladder	99 (20.0)
Viral etiology	
Yes	49 (9.93)
No	286 (58.01)
Not reported	158 (32.0)
Drainage or stent	
Yes	136 (27.5)
No	357 (72.4)
Overweight	
Yes	195 (39.5)
No	281 (56.9)
Not reported	17 (3.4)
CA 19–9 median (range) IU/mL	111 (0.60–400,000)
Within normal levels	148 (30.0)
>Normal levels	319 (64.7)
Staging	
Locally advanced	106 (21.5)
Metastatic	387 (78.4)
Not reported	9 (1.8)
NLR	
<3	204 (41.3)
≥3	244 (49.3)
Not reported	45 (9.1)
ECOG PS	
0–1	474 (96.1)
2	19 (3.8)
Steroids	
Yes	10 (2.0)
No	483 (97.9)
Analgesics	
Yes	44 (8.9)
No	449 (91.0)
B‐blockers	
Yes	84 (17.0)
No	409 (93.3)
Ace inhibitors	
Yes	45 (9.1)
No	448 (90.8)
Antihypertensives	
Yes	151 (30.6)
No	342 (16.8)
Metformin	
Yes	26 (5.2)
No	467 (94.7)
Insulin	
Yes	17 (3.4)
No	476 (96.5)
Pancrelipase	
Yes	10 (2.0)
No	483 (97.9)
Anticoagulants–antiplatelets	
Yes	30 (6.0)
No	463 (93.9)
Anxiolytics–antidepressants	
Yes	11 (2.2)
No	482 (97.7)
PPIs	
Yes	155 (31.4)
No	338 (68.5)
Levothyroxine	
Yes	27 (5.4)
No	466 (94.5)
Diuretics	
Yes	42 (8.5)
No	451 (91.4)
Statins	
Yes	71 (14.4)
No	422 (85.5)
Acetylsalicylic acid	
Yes	50 (10.1)
No	443 (89.8)
Vitamin D	
Yes	9 (1.8)
No	484 (98.1)
Ursodeoxycholic acid	
Yes	50 (10.1)
No	443 (89.8)

Abbreviations: NLR, neutrophil to lymphocyte ratio; PPIs, proton pump inhibitors; PS, performance status.

### 3.2 Clinical outcomes

At the data cutoff (June 30, 2023), median follow‐up was 8.8 months (95% CI: 7.8–9.8). Median OS was 14.8 months (95% CI: 12.7–29.1) and median PFS was 8.2 months (95% CI: 7.4–8.9).

The univariate analysis showed that patients who did not take steroids and who did not take analgesics had longer OS compared with those who took these medications (14.8 vs. 5.0 months; HR 0.13; 95% CI: 0.03–0.48; *p* = .002; 14.9 vs. 9.4 months; HR 0.40; 95% CI: 0.22–0.73; *p* = .003, respectively). The other concomitant medications tested did not show any impact on OS. ECOG PS 0–1 (HR 0.22; 95% CI 0.09–0.55; *p* = .001), CA 19–9 within the normal range (HR 0.64; 95% CI: 0.45–0.90; *p* = .01), NLR <3 (HR 0.43; 95% CI: 0.31–0.60; *p* < .0001), and locally advanced disease (HR 0.49; 95% CI: 0.34–0.71; *p* = .0002) were all associated with longer OS in the univariate analysis (Table 2).

**TABLE 2:** Univariate and multivariate analysis of OS and PFS.

Parameter	Overall survival (OS)						Progression free survival (PFS)					
	Univariate			Multivariate			Univariate			Multivariate		
	HR	95% CI	*P*	HR	95% CI	*P*	HR	95% CI	*P*	HR	95% CI	*P*
Sex												
M: 1	0.94						1					
F: 2	1	0.60–1.48	.80				1		.62			
ECOG PS												
2	1			1			1			1		
0–1	0.22	0.09–0.55	.001	1.03	0.48–2.18	.93	0.48	0.23–0.99	.04	0.91	0.45–1.81	.79
Age												
≤70	1						1					
>70	0.86	0.62–1.18	.36				0.8856	0.69–1.13	.32			
CA 19‐9												
>NV	1			1			1			1		
NV	0.64	0.45–0.90	.01	0.59	0.40–0.87	.009	0.70	0.54–0.91	.008	0.69	0.51–0.92	.01
NLR												
≥3	1			1			1			1		
<3	0.43	0.31–0.60	<.0001	0.47	0.32–0.69	.0001	0.64	0.50–0.83	.0008	0.72	0.55–0.96	.02
Locally advanced: 1 Metastatic: 2												
2	1						1			1		
1	0.49	0.34–0.71	.0002	0.47	0.28–0.78	.003	0.64	0.48–0.85	.002	0.65	0.45–0.92	.01
Steroids												
Yes	1			1			1			1		
No	0.13	0.03–0.48	.002	0.37	0.13–0.94	.04	0.38	0.15–0.97	.04	0.72	0.70–1.42	.19
Analgesics												
Yes	1			1			1			1		
No	0.40	0.22–0.73	.003	0.83	0.43–1.59	.58	0.52	0.33–0.82	.0055	0.91	0.57–1.47	.72
B‐blockers												
Yes	1						1					
No	1.31	0.87–1.95	.18				1.21	0.89–1.65	.20			
Ace inhibitors												
Yes	1						1					
No	1.09	0.64–1.86	.73				1.24	0.83–1.85	.27			
Antihypertensives												
Yes	1						1					
No	1.14	0.81–1.60	.44				1.40	1.08–1.81	.01			
Metformin												
Yes	1						1					
No	1.42	0.72–2.80	.30				1.12	0.65–1.90	.67			
Insulin												
Yes	1						1					
No	2.18	0.82–5.82	.11				0.54	0.23–1.26	.15			
Pancrealipase												
Yes	1						1					
No	1.88	0.55–6.37	.30				0.71	0.25–1.98	.51			
Anticoagulants–antiaggregants												
Yes	1						1					
No	1.22	0.68–2.20	.49				1.28	0.82–2.02	.26			
Anxiolytics and antidepressants												
Yes	1						1					
No	1.67	0.62–4.53	.30				1.15	0.50–2.63	.72			
PPI												
Yes	1						1					
No	0.80	0.57–1.14	.22				0.90	0.69–1.17	.43			
Levothyroxine												
Yes	1						1					
No	1.15	0.61–2.16	.65				0.54	0.23–1.26	.87			
Diuretics												
Yes	1						1					
No	0.67	0.37–1.20	.18				0.97	0.63–1.50	.90			
Statins												
Yes	1						1					
No	0.85	0.74–1.86	.49				0.92	0.77–1.52	.63			
Acetylsalicylic acid												
Yes	1						1					
No	0.79	0.46–1.35	.40				0.96	0.64–1.44	.86			
Vitamin D												
Yes	1						1					
No	17.276	0.4482–66.598	.42				0.51	0.16–1.60	.25			
Ursodeoxycholic acid												
Yes	1		.26				1		.85			
No	0.73	0.43–1.26					0.96	0.64–1.44				

Abbreviations: NLR, neutrophil to lymphocyte ratio; PPIs, proton pump inhibitors.

At the univariate analysis, patients who did not take steroids and who did not take analgesics had a longer PFS compared with those who took these medications (8.2 vs. 3.5 months; HR 0.38; 95% CI: 0.15–0.97; *p* = .04; 8.5 vs. 6.6 months; HR 0.52; 95% CI: 0.33–0.82; *p* = .005, respectively). The other concomitant medications did not show any impact on PFS. ECOG PS 0‐1(HR 0.48; 95% CI: 0.23–0.99; *p* = .04), CA 19‐9 within the normal range (HR 0.70; 95% CI: 0.54–0.91; *p* = .008), NLR <3 (HR 0.64; 95% CI: 0.50–0.83; *p* = .0008), and locally advanced disease (HR 0.64; 95% CI: 0.48–0.85; *p* = .002) were associated with longer PFS at the univariate analysis (Table 2).

**FIGURE 1:**
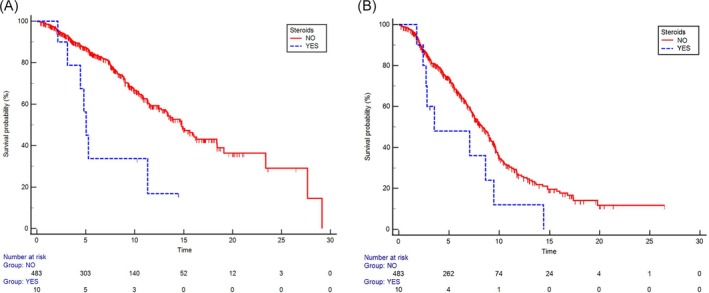
(A) Kaplan–Meier curves of OS in patients who did not take steroids and who did take steroids at a dose greater than 10 mg of prednisone daily or equivalent. (B) Kaplan–Meier curves of PFS in patients who did not take steroids and who did take steroids at a dose greater than 10 mg of prednisone daily or equivalent.

To better assess the impact of steroids and analgesics, a multivariate analysis was performed and confirmed that only steroids (prednisone >10 mg/day or equivalent) had an impact on survival (HR 0.37; 95% CI: 0.13–0.94; *p* = .04) (Table 2 and Figure 1A). As for PFS, no statistically significant results were observed for either steroids or opioids. However, a positive trend toward a better PFS was noted for patients who did not take steroids (HR 0.72; 95% CI: 0.70–1.42; *p* = .19) (Table 2 and Figure 1B).

In addition, 12 (2.4%) vs. 0 (0%) CR, 125 (25.8%) vs. 3 (30.0%) PR, 209 (43.2%) vs. 2 (20.0%) SD, and 98 (20.2%) vs. 5 (50.0%) PD were observed in the group that did not receive prednisone >10 mg daily or equivalent and in the group that received steroids, respectively (Table S1).

No differences were reported in terms of ORR, DCR between those who received steroids (prednisone >10 mg or equivalent) before the start of CGD and those who did not take steroids (*p* = 1.0 and *p* = .16, respectively) (Table S1). In terms of adverse events, there are several significant statistical differences among the groups. The following events were observed: rash (34 vs. 0; *p* = .007), itching (48 vs. 0; *p* < .0001), diarrhea (77 vs. 0; *p* < .0001), thrombocytosis (56 vs. 0; *p* < .0001), neutropenia (26 vs. 9; *p* = .04), and fatigue (47 vs. 7; *p* < .001). These results compare patients who did not receive prednisone at doses >10 mg daily to those who did receive prednisone at doses greater than 10 mg daily (or equivalent; Table S2). The characteristics of 10 patients who took steroids (prednisone >10 mg or equivalent) before the start of CGD are summarized in Table S3.

## 4 DISCUSSION

In the present analysis, we first reported the effects of concomitant medications on survival outcomes in patients with advanced BTCs treated with CGD, and we highlighted that patients who did not receive steroids (prednisone >10 mg/day or equivalent) before starting chemoimmunotherapy had a longer survival compared with those who received steroids.

To the best of our knowledge, this is the first analysis that highlighted the correlation between steroids and poorer clinical outcomes in a large population of patients with BTCs treated with chemotherapy plus durvalumab. In the TOPAZ‐1 trial, patients who had taken prednisone >10 mg/day or equivalent within 14 days before receiving the first dose of durvalumab were excluded from enrolment. In contrast, our real‐world data include few patients who received prednisone >10 mg/day or equivalent and durvalumab at the same time.

It is important to note that in the multivariate analysis, the association between steroid use and shorter OS was maintained despite the small number of patients taking steroids (prednisone >10 mg/day or equivalent) compared with the total number of patients, making the result even more robust. Among the 10 patients who received steroids, one had an ECOG PS 2, while nine had an ECOG PS 0–1 before starting CGD. The average duration of steroid treatment was 72 days, with the most common reasons for prescription being pain—particularly bone pain—as well as fatigue, decreased appetite, and asthenia. Of note, a significant difference remained also when adding the data of ECOG PS. After analyzing the multivariate data for ECOG PS, we confidently conclude that we have eliminated a significant confounder that might have explained the initial positive result in the univariate analysis, thus reinforcing the findings in the multivariate analysis. The univariate analysis showed a significantly shorter PFS in patients taking steroids at doses >10 mg/day, although this finding was not confirmed in the multivariate analysis, where only a trend was observed.

Our results align with existing literature on other cancers, particularly advanced non‐small‐cell lung cancer (NSCLC), where several retrospective studies have shown poorer outcomes, including lower response rates, PFS, and OS, in patients treated with immunotherapy while receiving steroid doses exceeding 10 mg/day.^22^ A meta‐analysis of 14 studies, both randomized and observational, included 5461 patients with NSCLC treated with immunotherapy (nivolumab/pembrolizumab) who also received steroids (prednisone or equivalents) at a dosage of ≥10 mg/day, for brain metastases, cancer‐related symptoms, best supportive care, or immune‐mediated adverse events (imAEs). The analysis confirmed that patients receiving immunotherapy and steroids showed poorer OS and PFS compared with those who did not receive steroids.^23^

The biological basis that explains these results is not fully understood, but the opposite effects on the immune system of immunotherapy and steroids may play a role. While immunotherapy activates and multiplies tumor‐targeting CD8+ T cells and increases pro‐inflammatory cytokines while reducing regulatory T cells, steroids have the opposite effect.^24^ In fact, steroids exert their anti‐inflammatory effects by reducing the expression of many pro‐inflammatory genes, such as prostaglandins and cytokines; moreover, steroids lead to immunosuppression by impairing IL2‐mediated effector T‐cell activation and increasing regulatory T cells. In the tumor microenvironment, steroids affect the release of tumor antigens, lymphocyte trafficking, and immune‐mediated tumor killing.^25^ One study conducted in a mouse model responsive to anti–PD‐1 treatment found that PD‐1 blockade enhanced neoantigen‐specific CD8+ T‐cell responses, which led to tumor regression. However, when immunotherapy was used concurrently with steroids, there was a reduction in low‐affinity memory CD8+ T cells, resulting in blunted antitumor responses.^26^ Similarly, other research has shown that both circulating CD4+ and CD8+ T cells were reduced, and tumor growth increased in mice treated with steroids alone or in combination with anti–PD‐1 therapy, ultimately diminishing the therapeutic efficacy.^26^

Our research has several limitations. It is a retrospective investigation with possible confounding factors in the included cohorts, such as the ECOG PS, which could be affected by missing data on comorbidities. Moreover, due to the multicentric nature of the study, PFS, ORR, and DCR data have to be contextualized, and differences in tumor assessment modalities and time points among different institutions have to be considered. In retrospective studies, PFS is significantly affected by the different timing of radiological reevaluations, which could alter the results of population PFS. We only assessed the intake of concomitant medications before the start of the treatment, and no data are available regarding the use of steroids for imAEs during treatment. In addition, we do not know how many days before starting CGD patients were taking concomitant medications. The data we have only refer to the first day of CGD. Finally, no objective and definitive threshold dose of steroids above which there is a clinically significant effect has been established. Despite this, a cutoff of 10 mg of prednisone per day is conventionally used. Doses >10 mg daily increase the risk of infection and reduce immune function.^27^ Arbor et al. showed that patients with NSCLC who received steroids at a dose of >10 mg daily of prednisone or equivalent had poorer survival outcomes compared with those using a dose of equal or less than 10 mg daily at the start of PD‐(L)1 blockade.^28^ Considering these limitations, further prospective studies are needed to confirm our results.

In conclusion, the timing and use of steroids before starting ICIs are important clinical considerations. If clinically appropriate, steroids should be avoided or minimized before treatment initiation. While managing oncologic symptoms such as cachexia or symptomatic brain metastases, or for palliative care, the use of steroids may be necessary. However, whenever possible, steroid‐sparing approaches should be implemented, considering the unfavorable outcomes linked to the concurrent use of steroids and immunotherapy for cancer‐related symptoms. In phase 3 studies, it is emphasized that if the use of cortisone is deemed essential, the dosage should be carefully reduced to 10 mg before initiating immunotherapy. Consequently, the prescription of corticosteroids should be judiciously limited, ensuring that dosages are maintained at the lowest effective levels possible to minimize potential side effects and optimize patient outcomes.

Our analysis suggests the importance of carefully balancing the risks and benefits of the use of steroids (prednisone at doses >10 mg/day or its equivalent) before initiating treatment for CGD in patients who are experiencing asthenia, poor appetite, or in general, to improve ECOG PS before starting immunotherapy.

## AUTHOR CONTRIBUTIONS

**Anna Saborowski:** Data curation. **Francesca Salani:** Conceptualization; data curation; writing – original draft; writing – review and editing. **Fabian Finkelmeier:** Data curation. **Mario Domenico Rizzato:** Data curation. **Silvia Camera:** Conceptualization; writing – original draft; writing – review and editing; data curation. **Tiziana Pressiani:** Data curation. **Federico Rossari:** Data curation. **Lorenzo Antonuzzo:** Data curation. **Frederik Peeters:** Data curation. **Ilario Giovanni Rapposelli:** Data curation. **Jessica Lucchetti:** Data curation. **Alessandro Parisi:** Data curation. **Oluseyi Abidoye:** Data curation. **Jin Won Kim:** Data curation. **Pircher Chiara:** Data curation. **Stefano Tamberi:** Data curation. **Chiara Gallio:** Data curation. **Guido Giordano:** Data curation. **Tomoyuki Satake:** Data curation. **Florian Castet:** Data curation. **Chiara Braconi:** Data curation. **Monica Verrico:** Data curation. **Alessandro Pastorino:** Data curation. **Aitzaz Qaisar:** Data curation. **Mario Scartozzi:** Data curation. **Changhoon Yoo:** Data curation. **Emiliano Tamburini:** Data curation. **Anna Diana:** Data curation. **Il Hwan Kim:** Data curation. **Gerald W. Prager:** Data curation. **Hong Jae Chon:** Data curation. **Marta Schirripa:** Data curation. **Antonio Avallone:** Data curation. **Jorge Adeva:** Data curation. **Ester Oneda:** Data curation. **Lukas Perkhofer:** Data curation. **Nuno Couto:** Data curation. **Nicola Personeni:** Data curation. **Ingrid Garajova:** Data curation. **Monica Niger:** Data curation. **Daniele Lavacchi:** Data curation. **Stephen L. Chan:** Data curation. **Ricardo Roque:** Data curation. **Mariam Grazia Polito:** Data curation. **Gian Paolo Spinelli:** Data curation. **Maria Grazia Rodriquenz:** Data curation. **Linda Bartalini:** Data curation. **Giada Grelli:** Data curation. **Matteo Landriscina:** Data curation. **Federica Lo Prinzi:** Conceptualization; writing – original draft; writing – review and editing; data curation. **Emanuela Di Giacomo:** Data curation. **Masafumi Ikeda:** Data curation. **Jeroen Dekervel:** Data curation. **Giovanni Farinea:** Data curation. **Antonio De Rosa:** Data curation. **Silvana Leo:** Data curation. **Giulia Tesini:** Data curation. **Rita Balsano:** Data curation. **Minsu Kang:** Data curation. **Giuseppe Tonini:** Data curation. **Tanios Bekaii‐Saab:** Data curation. **Vera Himmelsbach:** Data curation. **Alessandra Boccaccino:** Data curation. **Selma Ahcene Djaballah:** Data curation. **Sara Lonardi:** Data curation. **Lorenzo Fornaro:** Data curation; conceptualization; writing – original draft; writing – review and editing. **Cecilia Melo Alvim:** Data curation. **Arndt Vogel:** Data curation. **Gianluca Masi:** Data curation. **Andrea Casadei‐Gardini:** Conceptualization; data curation; writing – review and editing; writing – original draft. **Margherita Rimini:** Conceptualization; data curation; writing – original draft; writing – review and editing. **Lorenza Rimassa:** Conceptualization; data curation; writing – original draft; writing – review and editing.

## CONFLICT OF INTEREST STATEMENT

LR received consulting fees from AbbVie, AstraZeneca, Basilea, Bayer, BMS, Eisai, Elevar Therapeutics, Exelixis, Genenta, Hengrui, Incyte, Ipsen, IQVIA, Jazz Pharmaceuticals, MSD, Nerviano Medical Sciences, Roche, Servier, Taiho Oncology, Zymeworks; lecture fees from AstraZeneca, Bayer, BMS, Guerbet, Incyte, Ipsen, Roche, Servier; travel expenses from AstraZeneca; research grants (to Institution) from AbbVie, Agios, AstraZeneca, BeiGene, Eisai, Exelixis, Fibrogen, Incyte, Ipsen, Jazz Pharmaceuticals, Lilly, MSD, Nerviano Medical Sciences, Roche, Servier, Taiho Oncology, TransThera Sciences, Zymeworks.

ACG reports consulting fees from AstraZeneca, Bayer, BMS, Eisai, Incyte, Ipsen, IQVIA, MSD, Roche, Servier; lecture fees from AstraZeneca, Bayer, BMS, Eisai, Incyte, Ipsen, Roche, Servier; travel expenses from AstraZeneca; research grants (to Institution) from AstraZeneca, Eisai.

SLC serves as an advisory member for AstraZeneca, MSD, Eisai, BMS, Ipsen, and Hengrui, received research funds from MSD, Eisai, Ipsen, SIRTEX, and Zailab, and honoraria from AstraZeneca, Eisai, Roche, Ipsen, and MSD.

TP received consulting fees from Bayer, Ipsen, and AstraZeneca; institutional research funding from Roche, Bayer, and AstraZeneca; travel expenses from Roche.

CB received honoraria as speaker (Astrazeneca, Incyte, Servier) and consultant (Incyte, Servier, Boehringer Ingelheim, Astrazeneca, Tahio, Jazz), received research funds (Avacta, Medannex, Servier) and her spouse is an employee of Astrazeneca.

M. Ikeda reports honoraria from AstraZeneca, Chugai Pharma, Eisai, Incyte, Lilly Japan, MSD, Novartis, Ono Pharmaceutical, Takeda, Teijin Pharma, Nihon Servier, Taiho and research funding from AstraZeneca, Bayer, Bristol‐Myers Squibb, Chiome Bioscience, Chugai, Eisai, Eli Lilly Japan, Delta‐Fly Pharma, Invitae, J‐Pharma, Merck biopharma, Merus N.V., MSD, Novartis, Nihon Servier, Ono, Syneos Health, and Rakuten Medical.

GWP: Advisories and/or Speaker fees: Servier, Bayer, Roche, Amgen, Merck, MSD, BMS, Sanofi, Lilly, Astra Zeneca, Astellas, Pierre‐Fabre, Incyte, Arcus, CECOG.

F. F. has received travel support from Ipsen, AbbVie, AstraZeneca, and speaker's fees from AbbVie, MSD, Ipsen, AstraZeneca.

LP: Advisory role: AstraZeneca, Servier, Travel expenses: AstraZeneca, Ipsen.

GG: Consulting Fees: Astra Zeneca, MSD, Servier, Seagen, Bayer, Amgen, Novartis, Ipsen, BMS.

Travel Expenses: Astra Zeneca, Servier, Bayer, Novartis.

S. Leo reports research funding (to Institution) from Amgen, Astellas, Astra Zeneca, Bayer, Bristol‐Myers Squibb, Daiichi Sankyo, Hutchinson, Incyte, Merck Serono, Mirati, MSD, Pfizer, Roche, Servier; personal honoraria as invited speaker from Amgen, Astra Zeneca, Bristol‐Myers Squibb, Incyte, GSK, Lilly, Merck Serono, MSD, Pierre‐Fabre, Roche, Servier; participation in advisory board for Amgen, Astellas, Astra Zeneca, Bayer, Bristol‐Myers Squibb, Daiichi Sankyo, GSK, Incyte, Lilly, Merck Serono, MSD, Servier, Takeda, Rottapharm.

JD received consulting fees and/or speaker honoraria from Amgen, AstraZeneca, Bayer, BMS, Eisai, Need Inc., Ipsen, Lilly, MediMix, Merck, MSD, Novartis, Roche, and Servier.

JA received consulting fees from AstraZeneca, Jazz Pharmaceuticals, MSD, Roche, Servier, Taiho Oncology, Zymeworks; lecture fees from AstraZeneca, Roche, Servier; travel expenses from AstraZeneca, Roche, Servier.

AD Advisory Board: Amgen, Gentili, Invited Speaker: Eli Lilly, Novartis, Pfizer, Gentili, Amgen, Daiichi‐Sankyo, Roche, Gilead, Travel Support: Eli Lilly, Pfizer, Novartis, Ipsen, Gentili, Gilead, Editorial Collaboration: ACCMED.

GPS received advisory role/travel from Bayer, Roche, J&J, MSD, and Novartis.

MDR received honoraria as a speaker from Astrazeneca.

FC received speaker fees from AstraZeneca, Eisai, OncoSil, Roche, Rovi, and Servier and travel and accommodation from Roche and Servier.

MN received travel expenses from AstraZeneca, speaker honorarium from Accademia della Medicina, Incyte, and Servier; honoraria from Sandoz, Medpoint SRL, Incyte, AstraZeneca, and Servier for editorial collaboration. Consultant; honoraria from EMD Serono, Basilea Pharmaceutica, Incyte, MSD Italia, Servier, AstraZeneca and Taiho.

All remaining authors have declared no conflicts of interest.

## ETHICS STATEMENT

The study was conducted in accordance with the Declaration of Helsinki and the protocol was approved by the Ethics Committee of each institution involved in the project. Under the condition of retrospective archival tissue collection and patients' data anonymization, our study was exempted from the acquisition of informed consent from patients by the institutional review board. The Ethical Review Board of each Institutional Hospital approved the present study. This study was performed in line with the principles of the Declaration of Helsinki.

## Supporting information


**Data S1.** Supporting Information.

## Data Availability

The data that support the findings of this study are available from the corresponding author upon reasonable request.
