# Supplementary material for: Negative impact of corticosteroid use on outcome in patients with advanced BTCs treated with cisplatin, gemcitabine, and durvalumab: A large real‐life worldwide population
Source: Int J Cancer. 2025 Jul 11;157(10):2092–102. doi: 10.1002/ijc.70009 (PMC12439076; doi:10.1002/ijc.70009)
Supplement: Supplementary file 1 — Data S1. Supporting Information. [file IJC-157-2092-s001.pdf]

Negative impact of corticosteroid use on outcome in patients with advanced BTCs treated with cisplatin, gemcitabine and durvalumab: A large real-life worldwide population

Federica Lo Prinzi, Francesca Salani, Silvia Camera, Mario Domenico Rizzato, Anna Saborowski, Lorenzo Antonuzzo, Federico Rossari, Tomoyuki Satake, Frederik Peeters, , Tiziana Pressiani, Jessica Lucchetti, Jin Won Kim, Oluseyi Abidoye, Ilario Giovanni Rapposelli,Chiara Gallio, Stefano Tamberi, Fabian Finkelmeier, Guido Giordano, Pircher Chiara, Hong Jae Chon, Chiara Braconi, Aitzaz Qaisar, Alessandro Pastorino, Florian Castet, Emiliano Tamburini, Changhoon Yoo, Alessandro Parisi, Anna Diana, Mario Scartozzi, Gerald W. Prager, Antonio Avallone, Marta Schirripa, Il Hwan Kim, Lukas Perkhofer, Ester Oneda, Monica Verrico, Nuno Couto, Jorge Adeva, Stephen L. Chan, Gian Paolo Spinelli, Nicola Personeni, Ingrid Garajova, Maria Grazia Rodriquenz, Silvana Leo, Cecilia Melo Alvim, Ricardo Roque,Mariam Grazia Polito,Emanuela Di Giacomo, Giovanni Farinea, Linda Bartalini, Giada Grelli, Antonio De Rosa, Daniele Lavacchi, Masafumi Ikeda, Jeroen Dekervel, Monica Niger, Rita Balsano, Giuseppe Tonini, Minsu Kang, Giulia Tesini, Alessandra Boccaccino, Vera Himmelsbach, Matteo Landriscina , Selma Ahcene Djaballah, Tanios Bekaii-Saab,Lorenzo Fornaro, Gianluca Masi, Arndt Vogel, Sara Lonardi, Margherita Rimini, Lorenza Rimassa, Andrea Casadei-Gardini

Supplementary Tables Legends

Supplementary Table 1: ORR and DCR in patients who did not take steroids and who did take steroids at a dose greater than 10 mg of prednisone daily or equivalent

Supplementary Table 2: Comparison of adverse events in the two groups of patients selected according to toxicity grade 0-2 or >2 toxicity in accordance with the Common Terminology Criteria for Adverse Events v5.0 (CTCAE)

Supplementary Table 3: Features of ten patients who did take steroids at a dose greater than 10 mg of prednisone daily or equivalent. CGD. cisplatin, gemcitabine and durvalumab

Supplementary Table 1: Objective response rate and disease control rate in patients who did not take steroids and who did take steroids at a dose greater than 10 mg of prednisone daily or equivalent. ORR: objective response rate; DCR: disease control rate; CR: complete response; PR: partial response; SD: stable disease; PD: progression disease.

	**No Steroids** N=483	**Yes Steroids** N=10	**p**
Best Overall Response			
CR	12(2.4)	0(0)	
PR	125(25.8)	3(30.0)	
SD	209(43.2)	2(20.0)	
PD	98(20.2)	5(50.0)	
ORR	137(28.3)	3 (30.0)	1.00
DCR	346 (71.6)	5(50.0)	0.16

Supplementary Table 2: Comparison of adverse events in the two groups of patients selected according to toxicity grade 0-2 or >2 toxicity in accordance with the Common Terminology Criteria for Adverse Events v5.0 (CTCAE). ALT: alanine transaminase.

**Adverse Event**	**No Steroids<br>N=483<br>N (%)**	**Yes Steroids<br>N=10<br>N(%)**	**p**
**Colitis**<br>yes<br>1<br>=/>2	5(1.0)<br>5(1.0)<br>0(0)	0(0)<br>0(0)<br>0(0)	1.0
**Colangitis**<br>yes<br>1<br>=/>2	50(10.3)<br>4(0.8)<br>46(9.5)	0(0)<br>0(0)<br>0(0)	1.0
**Hypothyroidism**<br>yes<br>1<br>=/>2	24(4.9)<br>14(2.8)<br>10(2.0)	0(0)<br>0(0)<br>0(0)	1.0
**Hyperthyroidism**<br>yes<br>1<br>=/>2	9(1.8)<br>4(0.8)<br>5(1.0)	0(0)<br>0(0)<br>0(0)	0.33
**Rash**<br>yes<br>1<br>=/>2	34(7.0)<br>21(4.3)<br>13(2.6)	0(0)<br>0(0)<br>(0)	0.007
**Itching**<br>yes<br>1<br>=/>2	48(9.9)<br>36(7.4)<br>11(2.2)	0(0)<br>0(0)<br>0(0)	<0.0001
**Other immune-mediated toxicities**<br>yes<br>1<br>=/>2	27(5.5)<br>13(2.6)<br>14(2.8)	3(30)<br>1(10)<br>2(20)	0.8
**Diarrhea**<br>yes<br>1<br>=/>2	77(15.9)<br>56(11.5)<br>21(4.3)	0(0)<br>0(0)<br>0(0)	<0.0001
**Thrombocitosis**<br>yes<br>0-1	56(11.5)<br>47(9.7)	0(0)<br>0(0)	<0.0001

=/>2	9(1.8)	0(0)	
**Vomiting**<br>yes<br>1<br>=/>2	59(12.2)<br>43(8.9)<br>16(3.3)	3(30)<br>3(30)<br>0(0)	0.50
**Thrombocytopenia**<br>yes<br>1<br>=/>2	182(37.2)<br>91(18.8)<br>91(18.8)	4(40)<br>1(10)<br>3(30)	0.61
**Constipation**<br>yes<br>1<br>=/>2	17(3.5)<br>15(3.1)<br>2(0.41)	1(10)<br>1(10)<br>0(0)	0.93
**Fever**<br>yes<br>1<br>=/>2	18(3.7)<br>13(2.69)<br>5(1.0)	1(10)<br>1(10)<br>0(0)	0.82
**Leukopenia**<br>yes<br>1<br>=/>2	23(4.7)<br>6(1.24)<br>17(3.51)	1(10)<br>0(0)<br>1(10)	0.84
**Nausea**<br>yes<br>1<br>=/>2	27(5.5)<br>14(2.8)<br>13(2.6)	4(40)<br>2(20)<br>2(20)	0.99
**Neuropathy**<br>yes<br>1<br>=/>2	10(0.2)<br>8(1.6)<br>2(0.4)	0(0)<br>0(0)<br>0(0)	0.07
**ALT increased**<br>yes<br>1<br>=/>2	30(6.2)<br>23(4.7)<br>7(1.4)	1(10)<br>1(10)<br>0(0)	0.86
**Neutropenia**<br>yes<br>0-1<br>=/>2	26(5.3)<br>7(1.4)<br>19(3.9)	9(90)<br>1(10)<br>0(0)	0.04
**Anemia**<br>yes<br>1<br>=/>2	46(9.5)<br>24(4.9)<br>22(4.5)	5(50)<br>0(0)<br>5(50)	0.08

**Fatigue**<br>yes<br>1<br>=/>2	 47(9.7)<br>27(5.5)<br>20(4.1)	 7(70)<br>44(0)<br>3(30)	 <0.0001
**Other toxicity**<br>yes<br>1<br>=/>2	 5(1.0)<br>2(0.4)<br>3(0.6)	 2(20)<br>2(20)<br>0(0)	 <br>0.34

Supplementary Table 3: Features of ten patients who did take steroids at a dose greater than 10 mg of prednisone daily or equivalent. CGD: cisplatin, gemcitabine and durvalumab

**Patients**	**Type of steroid**	**Daily dose**	**Reason**	**Start of steroid**	**End of steroid**	**Start of CGD**	**Duration of steroid administration**	**Maintenance with durvalumab and number of cycles**
Patient 1	Dexamethasone	4mg	Pain-Radiotherapy on lymph node	1 March 2022	19 July 2022	22 April 2022	141 days	No
Patient 2	Dexamethasone	8mg	Bone pain	8 March 2022	21 April 2022	13 May 2022	45 days	No
Patient 3	Dexamethasone	4mg	Nausea	5 April 2022	20/05/2022	29 April 2022	46 days	No
Patient 4	Prednisone	25mg	Fatigue-bilirubin increase-decreased appetite	30 January 2023	11 April 2024	27 February 2023	71 days	Yes-5 cycles
Patient 5	Dexamethasone	4mg	Asthenia	11 May 2022	13 July 2022	21 June 2022	71 days	No
Patient 6	Prednisone	12.5mg	Nausea-vomiting	12 October 2023	21 February 2024	18 October 2023	139 days	No available data
Patient 7	Dexamethasone	4mg	Bone pain	27 May 2022	22 June 2022	3 June 2022	26 days	Yes-2 cycles

Patient 8	Prednisone	12.5mg	Pain	1 September 2022	1 February 2023	27 June 2022	144 days	No
Patient 9	Prednisone	12.5mg	Pain	20 June 2022	1 September 2023	29 August 2023	73 days	Yes-4 cycles
Patient10	Prednisone	37.5mg	Pain	11 July 2023	10 October 2023	04 October 2022	92 days	No
							Median duration of steroid administration: 72 days	
